# Internet addiction among school adolescents in Jeddah-Saudi Arabia

**DOI:** 10.1186/s42506-024-00157-9

**Published:** 2024-05-27

**Authors:** Ahmad Ismail, Omar Alamri, Abd-Alhadi Hassan, Alaa Hafiz, Mohammad Othman, Dena Atallah, Mashael F. Dewan

**Affiliations:** 1 Department of Nursing, Fakeeh College for Medical Sciences, Jeddah, 21461 Saudi Arabia; 2 Faculty of Nursing, King Abdulaziz University, Jeddah, Saudi Arabia; 3 Department of Medicine, Fakeeh College for Medical Sciences, Jeddah, 21461 Saudi Arabia

**Keywords:** Internet addiction, Adolescents, Saudi Arabia

## Abstract

**Background:**

Internet addiction is increasing among adolescents worldwide. There is a lack of research assessing internet addiction and factors contributing to it among adolescents in Jeddah city. The current study aimed to assess the rate of internet addiction among adolescents in Jeddah, Saudi Arabia, and the potential factors associated with it.

**Methods:**

A cross-sectional online survey, hosted by SurveyMonkey, was used to capture data on internet use from 462 adolescents aged 12–18 years between March and May 2022. Young’s Internet Addiction Scale was used to assess the degree of internet addiction as perceived by adolescents. Multiple linear regression analysis was used to identify possible predictors of internet addiction among adolescents in Jeddah.

**Results:**

The mean age of the participants was 15.5 ± 1.9 years. The majority were females (75%) from public schools (63%), spent an average of six hours on the internet daily, owned smartphones (98%), accessed the internet via a smartphone (94%), and used the internet for socializing (82%). Internet addiction mean score was 39.20 ± 15.20 out of 100. More than two-thirds of the participants had mild to moderate levels of internet addiction (68%). Significant predictors contributing to internet addiction were using the internet for socialization and playing online games. The more hours spent on the internet daily, the more the internet addiction was (*p* ≤ 0.05).

**Conclusions:**

The internet addiction rate is high among school adolescents in Jeddah. The majority of high school adolescents had mild to moderate levels of internet addiction. Interventional multidisciplinary programs are needed to mitigate the factors that influence internet addiction.

## Introduction

Using the internet has increased in the last few years worldwide. The Internet has many uses and offers a chance to communicate with people without restriction. The Internet is used for completing work, education, paying bills, shopping, online banking, reading, writing emails, playing games, engaging in communication, and socialization. Internet use has increased with the increase in the popularity of social media platforms and the boom in online gaming which has led to an increase in the amount of time people spend online. Although the internet provides many advantages, spending too much time on the internet may lead to physical and psychological negative consequences [1–3].

Internet addiction is defined by the American Psychological Association (APA) as “a behavioral pattern characterized by excessive or obsessive online and offline computer use that leads to distress and impairment” [4]. Internet addiction disorder is indicated by too much internet use that compromises the completion of daily activities and may even harm daily functions [5]. Research reveals that the internet is excessively being used by adolescents. American teenagers spend an average of six and a half hours on social media daily [6]. In addition, nearly 90% of teens use the internet in various ways, including social networking, blogging, chatting, instant messaging, uploading photos and videos, and gathering information by visiting websites [7].

Internet addiction harms adolescents’ life as well as their health (physical and psychological). It affects nearly every aspect of the life of adolescents. Among the reported physical health consequences were overweight and obesity, compromised physical fitness, and problems in the vision and the musculoskeletal system [8]. The psychosocial negative outcomes include stress, anxiety, depression, increased loneliness, and low self-esteem. Furthermore, internet addiction may lead to insomnia, poor performance in school, a perception of a low quality of life, and strained relationships with family and friends [9–11].

The prevalence of internet addiction in Saudi Arabia was measured across time, populations, and cities. It was as low as 5% in 2010 among secondary school students in the capital city of Saudi Arabia (Riyadh) [12] and 51% among female university students at El Jouf University in 2017. This higher rate was explained by the fact that university students utilize the internet to alleviate their stress arising from academic responsibilities [1]. The prevalence of internet addiction was 6% among university medical students at Taibah University in Madinah in 2020 [13]. Another recent research found that 5% of school students in Saudi Arabia (grades 7-12) were addicted to electronic games in Al-Qassim province; which is considered as one kind of internet addiction [14].

Research conducted in Saudi Arabia identified some factors associated with the increased rate of internet addiction. Firstly, there has been a notable increase in internet coverage throughout Saudi Arabia over the past decade. Secondly, the majority of both youths and adults in the country own smartphones and engage in internet usage for more than two hours daily. Additionally, the use of social media applications, such as WhatsApp, Snapchat, and Twitter, has experienced a surge in terms of both quantity, content, and overall popularity. Lastly, the intense heat experienced during the summer months may compel individuals to stay indoors during daytime, potentially leading to increased internet usage [3, 15]. However, no study has yet been conducted to assess the rate of internet addiction and factors contributing to internet addiction among adolescents in Jeddah city. Therefore, this research aims to assess the rate of internet addiction and identify possible factors associated with internet addiction among adolescents in the second-largest city in Saudi Arabia, Jeddah city.

## Methods

### Study design

This research used a cross-sectional design to collect data from 462 Adolescents. Data collection took place between March and May 2022 in Jeddah city- Saudi Arabia. Jeddah city is the second largest populated city in Saudi Arabia after the capital city (Riyadh).

### Target population

Adolescents (aged 12-18 years old) from six schools in Jeddah, Saudi Arabia participated in this study. The following were the inclusion criteria for the study participants: 1) the participant must be an adolescent (12 to 18 years old), 2) able to read and write in Arabic as the survey instrument was administered in Arabic, and 3) the participant must have internet access.

### Study setting

The total number of schools in Jeddah city is 2028 [16]. The research was focused on intermediate and high schools in Jeddah City. Six schools were selected using convenience sampling based on their willingness to participate.

### Sample size

The minimum sample size was calculated using the Slovin Formula [17]. The *n* = N / (1+Ne^2), Where n is the targeted sample size, N is the population size, and e is the margin of error (0.05). The population size in Jeddah city based on the City of Jeddah Municipality is 3.4 million people [18]. Fourteen percent of the population are adolescents [19, 20]. Therefore, the estimated number of adolescents in Jeddah city is four hundred seventy-six thousand (476,000). The targeted sample size was thus 476,000/ (1+476,000* 0.05^2) = 400 participants.

### Data collection tools

An online survey hosted by SurveyMonkey was designed to capture data in three main sections:

1. Demographic items including age, gender, and school type.
2. Internet use-related questions are seven items asking about the device used to access the internet, the source of the internet connection, having a smartphone, time spent on the internet daily, having internet at home, access to the internet outside the home, and the purpose of internet usage.
3. The Arabic version of Young’s Internet addiction Scale [21]. It is one of the most utilized screening instruments for internet addiction, developed by Young [22]. It consists of 20 statements; respondents answer item questions on a 6-point scale. Items are rated on a scale from 0 to 5 (0 = Not Applicable, 1 = Rarely, 2 = Occasionally, 3 = Frequently, 4 = Often, 5 = Always) reflecting the time spent and the consequences of Internet use on life. A total score that ranges from 0 to 30 points was considered to reflect a normal level of internet usage; scores of 31 to 49 indicated the presence of a mild level of internet addiction; 50 to 79 indicated the presence of a moderate level of addiction, and scores of 80 to 100 indicated a severe dependence upon the internet [22]. The internal consistency of the Arabic version of Young’s Internet addiction scale was excellent (0.92). The exploratory factor analysis indicated that the scale is one model fit [21]. The internal consistency of the currently used scale was high (Cronbach’s Alpha = 0.87).

### Statistical analysis

The Statistical Package for Social Sciences (SPSS) version 24 (Armonk, NY: IBM Corp.) was used to analyze the data. Qualitative data were described using the number and percent while numerical data were represented using mean and standard deviation (SD). The value of significance was set at *p*< 0.05. Multivariate analysis using the multiple linear regression model was used to identify possible predictors for internet addiction. The dependent variable is the internet addiction (continuous variable), and the independent variables are age, gender, school type, device used to access internet, connection type for internet, owning a Smartphone, availability of home internet, availability of internet outside the home, purpose of internet use, and number of daily hours spent on the internet. Categorical variables were dummy coded. Data of the dependent variable (internet addiction) tended to follow the normal distribution. Multicollinearity was tested using the tolerance test for all independent variables in the regression model. It ranged from 0.10 to 0.91, indicating no evidence of multicollinearity.

## Results

Four hundred sixty-two participants completed the survey out of 616 who started the survey. Data screening revealed no more than 10% missing in any item. Missing data were treated using pairwise deletion. The mean age of the participants was 15.5± 1.9 years. Most of the participants were females (75%) and from public schools (63%) (Table 1).

**Table 1:** Demographic characteristics of the studied school adolescents in Jeddah, Saudi Arabia, 2022 (*n* = 462)

Characteristics	Frequency	%	Mean	SD
Age (Years)			15.5	1.9
Gender				
Female	347	75.0		
Male	115	25.0		
School				
Public	291	63.0		
Private	171	37.0		

The participants spent an average of six hours on the internet daily (6.33 ± 2.6), owned a Smartphone (98%), accessed the internet via a Smartphone (94%) using home Wi-Fi (68%), used the internet for socializing (82%), and had the chance to access the internet outside the home (77%) (Table 2).

**Table 2:** Internet use among the studied school adolescents in Jeddah, Saudi Arabia, 2022 (*n* = 462)

Internet use	Frequency	%	Mean	SD
Daily hours on the Internet			6.33	2.6
Own Smartphone				
Yes	452	98.0		
No	10	2.0		
How to access the Internet				
Smartphone	432	93.5		
Laptop	10	2.0		
Desktop computer	7	1.5		
Tablet	13	3.0		
Internet source				
Wi-Fi at home	316	68.0		
Mobile data	4	1.0		
Wi-Fi at school	142	31.0		
Internet at home				
Yes	447	97.0		
No	15	3.0		
Internet access outside home				
Yes	356	77.0		
No	105	23.0		
Purpose of Internet use				
Social networks	380	82.0		
Online gaming	28	6.0		
Watching video	30	7.0		
Online learning	24	5.0		

Internet addiction mean score was 39.20 ± 15.20. The highest scoring items of the internet addiction scale were “How often do you fear that life without the internet would be boring, empty, and joyless?” and “How often do you find that you stay online longer than you intended?” (3.02 ± 1.39 and 2.95 ±1.24 respectively) (Table 3).

**Table 3:** Young’s Internet Addiction Scale Item Responses of the studied school adolescents in Jeddah, Saudi Arabia, 2022 (*n* = 462)

Item	Min	Max^a^	Mean	SD
How often do you find that you stay online longer than you intended?	0	4	2.95	1.24
How often do you neglect household chores to spend more time online?	0	4	2.05	1.38
How often do you prefer the excitement of the internet to intimacy with your partner?	0	4	2.29	1.41
How often do you form new relationships with fellow online users?	0	4	1.98	1.70
How often do others in your life complain to you about the amount of time you spend online?	0	4	1.66	1.32
How often do your grades or schoolwork suffer because of the amount of time you spend online?	0	4	1.71	1.41
How often do you check your WhatsApp, Facebook or e-mail before something else that you need to do?	0	4	1.48	1.41
How often does your job performance or productivity suffer because of the internet?	0	4	1.55	1.31
How often do you become defensive or secretive when anyone asks you what you do online?	0	4	2.42	1.46
How often do you block out disturbing thoughts about your life with soothing thoughts of the internet?	0	4	1.63	1.36
How often do you find yourself anticipating when you will go online again?	0	4	2.16	1.46
How often do you fear that life without the internet would be boring, empty, and joyless?	0	4	3.02	1.39
How often do you snap, yell, or act annoyed if someone bothers you while you are online?	0	4	1.97	1.63
How often do you lose sleep due to late-night log-ins?	0	4	1.83	1.41
How often do you feel preoccupied with the internet when off-line, or fantasize about being online?	0	4	1.45	1.26
How often do you find yourself saying “just a few more minutes” when online?	0	4	2.11	1.47
How often do you try to cut down the amount of time you spend online and fail?	0	4	2.15	1.46
How often do you try to hide how long you’ve been online?	0	4	1.61	1.44
How often do you choose to spend more time online over going out with others?	0	4	1.44	1.59
How often do you feel depressed, moody, or nervous when you are off-line, which goes away once you are back online?	0	4	1.77	1.44
The mean total score			39.20	15.20

^a^The maximum response of the participants was 4 (None scored 5 as described in the original scale)

More than two thirds of the participants had mild to moderate levels of internet addiction (68.2%). (43.5% had mild addiction to the internet, and 25% had a moderate level of internet addiction) Nearly 32% had normal internet use, (Table 4).

**Table 4:** Internet Addiction Level among studied school adolescents, Saudi Arabia, 2022 (*n* = 462)

Addiction level	Frequency	%
Normal	146	31.6
Mild level of internet addiction	201	43.5
Moderate level of addiction	114	24.7
Severe dependence upon the internet	1	0.2

The overall regression model was statistically significant (R^2^ = 0.32, F = 5.3, *p* <0 .05). Important predictors associated with increased internet addiction were using the internet to access social media platforms (B = 0.59, *p* = 0.01), using the internet to play online games (B= 0.63, *p*= 0.02), and spending more time on the internet daily (B= 0.14, *p*= 0.01). Using the internet for online learning was associated with a decrease in the internet addiction rate (B= -1.66, *p*= 0.01) (Table 5).

**Table 5:** Summary of multiple linear regression analysis of key factors associated with internet addiction

Factor	B	*t*	*P*	*95% CI*	
				Lower	Upper
Age	0.00	-0.01	0.99	-0.07	0.07
Female	-0.12	-0.86	0.39	-0.38	0.15
Male^a^	0				
Public school	-0.32	-0.73	0.47	-1.20	0.56
Private school^a^	0				
Use Smartphone to access the internet	0.07	0.22	0.83	-0.60	0.74
Use laptop to access the internet	0.20	0.44	0.66	-0.70	1.10
Use desktop computer to access the internet	0.13	0.28	0.78	-0.79	1.06
Use home WIFI to access the internet	1.97	1.32	0.19	-0.96	4.90
Use mobile data to access the internet	0.90	1.19	0.24	-0.59	2.40
Had own Smartphone	0.46	1.52	0.13	-0.14	1.05
Had internet at home	0.15	0.37	0.71	-0.64	0.95
Had access to internet outside the home	0.20	1.63	0.11	-0.04	0.44
Use internet for social media	0.59*	2.70	0.01	0.16	1.01
Use internet to play games	0.63*	2.32	0.02	0.09	1.16
Use internet to watch online video	0.47	1.70	0.09	-0.08	1.03
Use internet for learning	-1.66*	-2.60	0.01	-2.90	-0.41
Hours spend on internet daily	0.14*	6.68	0.01	0.10	0.18

0^a^ Reference group

R^2^ = 0.32, F = 5.3, *p* < (0.05), *p < 0.05

## Discussion

This study found that internet addiction is relatively high among adolescents in Jeddah as a significant percentage (68%) of the participants in the present study hadmild to moderate internet addiction. The more time spent on the internet daily and the use of the internet for socializing and playing games, were associated with more internet addiction. The results of this study reported a higher internet addiction rate than previous studies conducted in Saudi Arabia [1, 12, 14]. Also, internet addiction in adolescents in this study is higher compared to the most reported rates worldwide. A systematic review reported that internet gaming addiction rates varied between 0.6 (in Norway) and 50% (in Korea) [23]. Studies from Europe show a lower rate of internet gaming addiction compared to our study. It was 3.5% in Germany and 12% in Italy [24, 25]. Our study includes any kind of internet addiction e.g., internet gaming, social media, and watching online video. Studies from Asia reveal similar results, a recent study which reviewed 38 studies on internet addiction among students in South Asia (Bangladesh, Nepal, Indonesia, Sri Lanka, Thailand, India, and Bhutan) between 2003 and 2016, revealed that the internet addiction rate ranged between zero and 47% [26]. In China, the internet addiction rate among middle school children was 13% [27], and it was 22% among South Korean adolescents [28].

The higher rate of internet addiction in the present study could be attributed to the COVID-19 pandemic mandating all educational institutions to use online learning instead of face-to-face. Consequently, most adolescents, who already owned their devices, relied more heavily on internet access for learning and completing school assignments, which increased the time they spend on the internet daily and decreased the time for face-to-face interaction. This is also supported by the multivariate analysis, which found that the more time spent on the internet daily, the higher the degree of internet addition was. Moreover, in response to the outbreak of COVID‐19, many measures were implemented to limit its spread, including staying at home which led to an increased use of the internet, and engaging in activities such as playing video games for entertainment [29, 30]. The COVID‐19 pandemic has also had a significant impact on mental health, prompting individuals to turn to the internet as a means of boosting their mental well-being and reducing stress [31], which in turn increases the risk of internet addiction. Previous research has identified several other factors contributing to internet addiction in Saudi Arabia. These include the expansion of internet coverage, with the majority of the population now having an internet connection. Furthermore, the majority of adolescents in Saudi Arabia own smartphones, and they use the internet for more than two hours daily, which further exacerbates the issue. The popularity of social media applications (e.g., WhatsApp, Snapchat, and Twitter) plays a significant role in this high rate, as well as the extreme heat during summer months, which forces adolescents to stay inside during the daytime and likely increases their internet use [3, 15]. In our study, we found that the female gender is not associated with higher internet addiction compared to males. This could be explained by COVID-19 measures mandating all adolescents, regardless of gender, to stay indoors and use the internet for socializing, playing games, watching videos and learning. However, other studies found that Saudi females are more addicted to the internet than males as Saudi female adolescents spend more time indoors compared to males [3, 15]. These studies were conducted before the COVID-19 pandemic.

The multivariate analysis conducted in this study identified many important factors that predict internet addiction among adolescents in Jeddah. Notably, the greater the daily duration of internet usage and engagement in socializing and gaming activities on the internet, the higher the likelihood of internet addiction. That is not surprising as most participants have smartphones, access the internet via smartphones, and spend an average of six hours on the internet daily for socialization and playing games. Consistent with previous research, our study found that adolescents who participated in this study spent a considerable number of hours on the internet daily (an average of six hours), own smartphones that are used to access the internet, and use the internet for socializing and playing games [3, 15]. A recent study from Vietnam reported that youth who use internet for socialization and playing games are more susceptible to internet addiction, possibly due to the increased familiarity and entertainment provided through internet use [32]. Another recent study from Qatar found that family and school environment are associated with internet addiction among adolescents [33]. These factors are, however, not addressed in our study. Therefore, future research should assess the impact of family and school factors on internet addiction in adolescents to provide a more comprehensive understanding of the issue.

### Limitations

This study has two main limitations that affect the generalizability of the results. First, this study used self-reporting where participants may respond based on their perception, often affected by personal bias as people try to provide socially acceptable responses rather than more truthful responses. The second is that the sample was collected from some intermediate and high schools in Jeddah using convenience sampling, and so the answers given may not be representative of the views of the general population of adolescents in Jeddah. In addition, this study did not study the scholastic achievement of the students.

## Conclusion

Internet addiction is widespread among adolescents in Jeddah city. Interventional multidisciplinary programs are needed to increase awareness of adolescents, parents, and schoolteachers in Jeddah about internet addiction. Those programs should provide possible solutions that help adolescents develop self-control measures for such issues. Future research should focus on identifying more important factors affecting adolescent internet addiction in Saudi Arabia. The model for such research should include the family, community, physical health, and psychological health factors of the participants. Future research is also needed to examine the effect of internet use and addiction on the psychological health of teens in Saudi Arabia.

## Data Availability

The dataset is available upon request.

## Abbreviations

APA: American Psychological Association
QR: Quick Response
SPSS: Statistical Package for Social Sciences
IRB: Institutional Review Board
N: Number
SD: Standard Deviation
COVID: Coronavirus disease
